# Virulence factors analysis and antibiotic resistance of uropathogenic *Escherichia coli* isolated from patients in northeast of Iran

**Published:** 2020-06

**Authors:** Mahdis Ghavidel, Tahere Gholamhosseini-Moghadam, Kimiya Nourian, Kiarash Ghazvini

**Affiliations:** 1 Antimicrobial Resistance Research Center, Mashhad University of Medical Sciences, Mashhad, Iran; 2 Department of Microbiology and Virology, School of Medicine, Mashhad University of Medical Sciences, Mashhad, Iran; 3 Department of Veterinary Medicine, Shiraz University, Shiraz, Iran; 4 Department of Veterinary Medicine, Ferdowsi University of Mashhad, Mashhad, Iran

**Keywords:** Urinary tract infections, *Escherichia coli*, Virulence factor, Multiplex polymerase chain reaction, Antibacterial drugs resistance

## Abstract

**Background and Objectives:**

*Escherichia coli* is known to be the pathogen commonly isolated from those infected with urinary tract infections (UTIs). The aim of this study was to investigate the presence of *E. coli* virulence genes and antibiotics’ resistance pattern among clinical isolates in the Northeast of Iran. Relationships between virulence genes and antimicrobial resistances were studied as well.

**Materials and Methods:**

Three hundred isolates of *E. coli* were isolated from patients with UTIs that referred to Ghaem and Imam Reza hospitals (Mashhad, Iran) during August 2016 to February 2017. A multiplex PCR was employed to amplify the genes encoding pyelonephritis associated pili *(pap)*, S-family adhesions *(sfa)*, type1fimbriae *(fimH)* and aerobactin *(aer)*. Disk diffusion test was performed to test the susceptibility of isolates to β-lactams, aminoglycosides, cephalosporins, quinolone, fluoroquinolones, carbapenems and trimethoprim-sulfamethoxazole.

**Results:**

The PCR results identified the *fimH* in 78.4%, *aer* in 70.5%, *sfa* in 13.6% and the *pap* in 8.2% of isolates. The rates of antibiotic resistance of the isolates were as follows: 64.7% resistant to cephalosporins, 34% to trimethoprim-sulfamethoxazole, 31% to fluoroquinolones, 15.3% to aminoglycosides, 13.3% to β-lactams, 7.8% to quinolones and 4.4% to carbapenems. Significant relationships existed between *pap* and *aer, pap* and *sfa, aer* and fluoroquinolones also *pap* and cephalosporins.

**Conclusion:**

*fimH* and *aer* were found in > 50% of isolates suggesting the importance of both genes in UPEC. The majority of isolates had *fimH* as adhesion factor for colonization. Determining antibiotic resistance patterns in specific geographical areas is necessary for appropriate treatment of urinary tract infection. The high rate of resistance to cephalosporins is most likely due to incorrect drug administration.

## INTRODUCTION

Being among the most prevalent microbial diseases, urinary tract infections (UTIs) can affect people of any age. There are different classifications for UTIs: healthcare associated urinary tract infections (HAUTI) and community associated urinary tract infections (CAUTI) (1).

UTIs can also be categorized into uncomplicated UTIs which mostly infect healthy individuals or those with no history of structural or neurological urinary tract abnormalities (2, 3) and complicated UTIs which involve factors attenuating urinary tract or the host’s defense system including situations like urinary tract obstruction, urinary retention due to neurological condition, suppressed immune system, renal failure, kidney transplant, gestation and the existence of foreign bodies such as calculi, drainage devices or indwelling catheters (4, 5).

Depends on the site of infection, UTIs are comprised of cystitis (bladder infection), pyelonephritis (kidney infection), prostatitis (prostate infection) and urosepsis, although bacteriuria is considered a probable typical symptom in all UTIs (6).

Uropathogenic *Escherichia coli* (UPEC) is considered as the dominant microorganism causing UTI. The microorganisms responsible for causing UTI are either Gram-negative or Gram-positive bacteria including *Escherichia coli, Proteus mirabilis, Klebsiella* species *(Klebsiella pneumoniae), Serratia marseciens, Citrobacter* species, *Gardnerella vaginalis, Pseudomonas aeruginosa, Enterobacter* species *(Enterococcus faecalis), Streptococcus* group B (GBS), *Staphylococcus* species *(Staphylococcus saprophyticus), Staphylococcus aureus, Mycoplasma* species, *Urea plasma* species and *Candida* spp. (6–9).

Depending on which virulence factors are expressed, UPEC’s ability of causing symptomatic UTIs will be affected. The major virulence factors of *E. coli* are either associated with the surface of the bacterial cell or secreted and exported to their site of action. Type 1 fimbriae (fimH), pyelonephritis– associated pilus (pap), and S fimbrial adhesion (sfa) are the adhesion factors which boost colonization through various processes.

Since type 1 fimbriae is expressed both in pathogenic and commensal isolates, it is hard to resolve its role in human diseases. In fact, the frequency of *fimH* gene would not alter if the isolates causing UTI have a high or low virulence. Although it has been proven that P fimbriae are responsible for the increase in early colonization of tubular epithelium by some mechanisms concerning inter bacterial binding and biofilm formation, type 1 fimbriae mediate the colonization of the center of tubule. P fimbriae, encoded by *pap* (pyelonephritis–associated pilus) operon, are the most significant mannose–resistant adhesions expressed by *E. coli* and will result in extra-intestinal infections (10).

Furthermore, S fimbrial adhesion encoded by *sfa* genes is considered a virulence factor. A recently documented virulence factor in UPEC isolates is aerobactin, a bacterial siderophore encoded by *aer* gene (11, 12).

The aim of this study was to determine the most prevalent virulence gene among *fimH, pap, sfa* and *aer* in *E. coli* isolated from patients with urinary tract infection and also to demonstrate antimicrobial susceptibility test (AST) using disk diffusion (Kirby–Bauer method) in northeast of Iran. Besides, the probable relationships among virulence factors and also between virulence factors and resistance to antimicrobial agents have been investigated.

## MATERIALS AND METHODS

### Bacterial isolates.

Three hundred *E. coli* strains were isolated from patients affected by UTIs from 17 different sections of Ghaem and Imam Reza hospitals in Mashhad, Iran, from August 2016 until February 2017.

To examine the collected urine samples, each was inoculated in EMB agar, MacConkey agar and Blood agar (Merk, Germany). Plates were incubated for 48 hours at 37°C. When *E. coli* isolates were confirmed after the cultures were proven positive for UTI (10^3^–10^5^ cfu/mL), the colonies were tested using Gram staining and related standard biochemical and microbiological analysis (including IMViC, SIM, etc.). The samples were stored at −70°C in Tryptic Soy Broth (TSB) medium supplemented with 10% glycerol.

### DNA extraction.

A single colony of each isolate was suspended in 100 μL distilled water, boiled for 10 minutes and then centrifuged at 10,000 × g for 10 minutes. The supernatants were collected carefully and used as template DNA for PCR (13).

### Multiplex PCR for virulence factors.

To amplify the genes which encode pyelonephritis associated pili (*pap* genes), S-family adhesions (*sfa* gene), type-1fimbriae ( *fimH* gene) and aerobactin (*aer* gene), a multiplex polymerase chain reaction (PCR) was employed. The primers used in the present study were the same as described by Baholo et al. (14).

The amplification reactions were carried out in a 50 μL reaction volume containing 5 μL 10 × PCR buffer, 5 mM dNTPs, 25 mM MgCl_2_, 5U of Taq DNA polymerase, 0.5 μM of each of the virulence gene-specific primers and 5 μL of template DNA using a thermal cycler (Astech, Ireland).

The amplification conditions included 32 cycles of a denaturation step at 94°C for 30 seconds, primer annealing at 54°C for 30 seconds and extension at 72°C for 1 minute. The extension time was ramped for an additional 3 seconds per cycle and a final extension step of 5 min at 72°C was performed. The PCR products were run in 1.5% agarose gel electrophoresis and gel was stained with green viewer (14).

### Antimicrobial susceptibility testing.

Antimicrobial resistance of *E. coli* isolates was investigated using disk diffusion on Mueller-Hinton agar plates (Merck, Germany) according to the Clinical and Laboratory Standards Institute guidelines, version 2016. The tested antimicrobials (Mast Companies, UK) were: ampicillin (10 μg), gentamicin (10 μg), amikacin (30 μg), cefazolin (30 μg), cefepime (30 μg), ceftazidime (30 μg), cefuroxime (30 μg), nalidixic acid (30 μg), ciprofloxacin (5 μg), norfloxacin (10 μg), imipenem (10 μg), meropenem (10 μg) and trimethoprim-sulfamethoxazole (12.5/23.75 μg). The quality control organism was *E. coli* ATCC 25922.

### Statistical analysis.

Data analysis was performed using SPSS software (SPSS 22). The relationship between different virulence factors and antimicrobial resistance was analyzed by Pearson Chi-squares teat or fisher exact test. P value of less than 0.05 was considered significant.

## RESULTS

The average age of the patients was 47, with the oldest being a 93-year-old man and the youngest patients were 11 boys and 7 girls below one year old. Among 300 isolates, 193 were isolated from females (64.3%), while 107 isolates (35.7%) were obtained from males.

### Multiplex PCR.

**Fig. 1.**
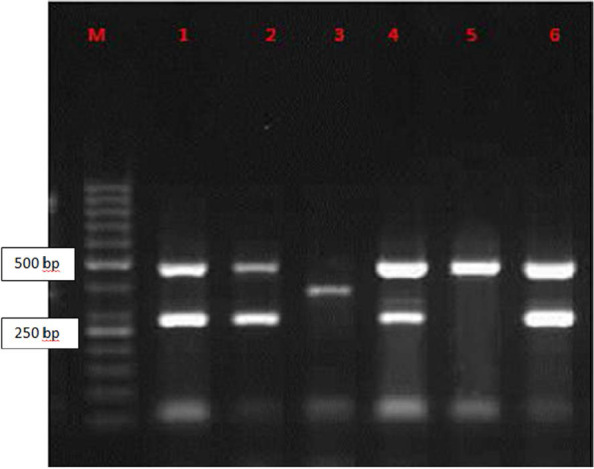
Electrophoresis of PCR product with different virulence patterns on 1.5% Agarose Gel; (a) Lanes M: 50-bpDNA ladder as the molecular size marker; lane1, 2, 6; UP7 (*aer* + *fimH*), lane 3; UP3 *(sfa)*, lane 4; UP12 (*aer* + *pap* + *fimH*), lane5; UP4 *(fimH)*.

The presence of virulence factors were investigated by PCR (Fig. 1). Among 300 isolates, *fimH* was found in 235 isolates (78.4%), *aer* in 212 (70.5%), *sfa* in 41 (13.6%) and *pap* in 25 (8.2%). Ten isolates did not have any virulence factors (3.33%).

Considering these 300 isolates, 94 isolates (31.3%) carried only 1 virulence gene, 168 isolates (56%) carried 2 virulence genes and 28 isolates (9.33%) were carriers of 3 virulence genes. The studied isolates showed 15 different virulence patterns which are shown as urovirulence profile (UP) in Table 1. The most prevalent urovirulence profiles were UP7 ( *fimH* + *aer*) and UP4 *(fimH)*. Of 300 isolates, 142 (47.7%) carried *fimH* and *aer* and 58 isolates (19.5%) had only *fimH*. The least common virulence profiles were UP10 (*sfa* + *fimH*) and UP14 (*aer* + *pap* + *sfa*), which were found in one isolate.

**Table 1.** Virulence genes’ patterns identified among 300 *E. coli* isolates

**UP**	**Virulence pattern**	**Number of isolates**	**Percentage %**
UP1	*aer*	29	9.6
UP2	*pap*	3	1
UP3	*sfa*	4	1.3
UP4	*fimH*	58	19.5
UP5	*aer* + *pap*	3	1
UP6	*aer* + *sfa*	12	4
UP7	*are* + *fimH*	142	47.7
UP8	*pap* + *sfa*	3	1
UP9	*pap* + *fimH*	7	2.3
UP10	*sfa* + *fimH*	1	0.3
UP11	*aer* + *sfa* + *fimH*	3	1
UP12	*aer* + *pap* + *fimH*	22	7.3
UP13	*pap* + *sfa* + *fimH*	2	0.7
UP14	*aer* + *pap* + *sfa*	1	0.3
UP15	none	10	3

UP: Urovirulence profile

### Antimicrobial susceptibility testing.

The highest resistance was detected in cephalosporins (n=194, 64.7%). The rates of resistance to the tested antibiotic were as follows: 102 isolates (34%) to trimethoprim-sulfamethoxazole, 93 isolates (31%) to fluoroquinolones, 46 isolates (15.3%) to aminoglycosides, 40 isolates (13.3%) to β-lactams and 23 isolates (7.8%) to quinolones. The lowest resistance was observed in carbapenems with only 4.4% (13 isolates).

### Statistical analysis of the associations between virulence factors and antimicrobial resistance.

The relationship between different virulence factors and between virulence factors and antimicrobial resistance were checked by chisquare test and then analyzed by fisher exact test (Table 2, 3).

**Table 2.** Relationship between virulence factors using chi square test

**Virulence factors**	***aer***	***pap***	***sfa***	***fimH***
*aer*	------	0.049^*^	0.643	0.594
*pap*	0.049^*^	------	0.005^*^	0.354
*Sfa*	0.643	0.005^*^	------	0
*fimH*	0.594	0.354	0	------

* P<0.05 is significant.

**Table 3.** Relationship between virulence factors and antimicrobial resistance using chi square test

	**β-lactam**	**Quinolones**	**Aminoglycosides**	**Fluoroquinolones**	**Trimethoprim-Sulfamethoxazole**	**Carbapenem**	**Cephalosporin**
*aer*	0.514	0.097	0.297	0.02^*^	0.639	0.061	0.876
*pap*	0.45	0.363	1	1	0.585	1	0.012^*^
*sfa*	1	0.101	1	0.348	0.067	0.603	0.171
*fimH*	0.621	0.757	0.639	1	0.723	0.657	0.597

* P<0.05 is significant.

The statistical analysis showed a significant relationship between *pap* and *aer* (p=0.049), suggesting that most *aer* positive isolates were *pap* negative. There was a significant association between *pap* and *sfa* (p=0.005), showing that most *pap* negative isolates were *sfa* negative. Moreover, a significant relationship was observed between *aer* and fluoroquinolones (p=0.02), which indicated that the isolates carrying *aer* genes are susceptible to fluoroquinolones. Another notable relationship was reported between *pap* and cephalosporins (p=0.012), which revealed that whenever *pap* is absent, the isolate would become resistant to cephalosporins.

## DISCUSSION

*Escherichia coli* is responsible for over 80% of urinary tract infections leading to renal failure in long term untreated conditions. UPEC pathogenicity can be affected by some virulence factors (15–17).

In our study, 300 *E. coli* isolates were isolated from 64.2% female patients and 35.7% from male patients. Farshad et al. and Staji et al. reported 62.5% of *E. coli* were isolated from females and 37.5% from males; in addition, Shah et al. detected 53% of *E. coli* from female patients and 37% from male ones. Likewise, Ghazvini et al. isolated 86% of *E. coli* from female patients and 14% from male patients. The reason for women to hold a higher percentage of UTIs might be ascribed to the fact that their urethra is not only shorter but also its orifice opening is closer to anus compared to men (18–21).

The adhesive subunit of type 1 fimbriae, *fimH*, was the most predominant virulence factor (78.4%), as Haghighatpanah et al. identified *fimH* in 74.4% of UPEC isolates (22). Among 12 studies which investigated the prevalence of *fimH*, 10 studies reported a high frequency of 64% to 100% for *fimH*. Only Hassan et al. and Bahalo et al. reported a low prevalence of 28% and 30% for *fimH*, respectively (14, 19, 22–32). In this study, most of the isolates carried *fimH* as fimbria factor; uropathogenic *E. coli* isolates, which cause a wide range of human UTIs, and infection following bacterial adhesion in which catch-bonds are significant. Furthermore, 0.1–2 mm long protein-aceous filaments located on the surface of the bacteria called type 1 pili act as mediators in the adhesion of bacteria to urothelial cells under flow conditions which is the initial phase of the infection (33).

The second most common factor was *aer* gene (70.5%). The highest rate for *aer* that has been reported in *E. coli* isolates is 90% in Tehran, Iran, while the lowest rate belongs to Shahr-e-kord, Iran, with only 12% of the isolates carrying the gene (14, 25); this data is based on 8 studies surveyed worldwide among which, four of them suggested the gene to have been present in over 50% of the isolates in Tunisia, Romania and Iran (26, 28, 29, 31, 34). Santo et al. reported a 76% rate for *aer* among 100 *E. coli* isolates in Brazil which is in agreement with our results (15).

S fimbrial adhesion factor *(sfa)* has been detected in 8 studies, 7 of which reported less than 50% *sfa* gene in the isolates (14, 20, 26, 29, 31). López-Banda et al. identified *sfa* in 74.1% of *E. coli* isolates in Mexico (27). Farshad et al. and Santo et al. reported an *sfa* rate of 14.6% and 19% respectively, in accordance with our results (13.6%) (15, 18).

The distribution of *pap* (8.2%) found among the studied isolates was lower than that of previously reported cases. Moreover, three studies identified *pap* to be > 50% and 13 studies detected it under 50% in isolates (14, 18–20, 26, 27, 29, 31, 32, 34–39). The highest and lowest rates for *pap* were 72% (Egypt) and 13% (Iran) (20, 32).

There seems to be a difference in the presence of different virulence factor genes such as *fimH, aer, sfa* and *pap* in *E. coli* isolates which can be attributed to regional geography, climate, diet, public health, customs and sampling techniques (40).

Firouzeh et al. discovered that 22.6% of the isolates carried only one virulence gene, 34.6% carried two virulence genes, 26% three of such genes and 3.5% four genes. In the present study, 31.3% of the isolates carried one virulence gene, 56% two genes and 9.33% three genes. The similarity between these two studies indicates that *E. coli* responsible for UTIs mostly carries two virulence genes (34). Various virulence factors are probably related to clinical signs of different diseases; *fimH* is associated with cystitis and descending infections, *pap* with pyelonephritis and ascending infections and *sfa, hly* or *aer* with primary sepsis (29).

Jalili et al. reported 20 different virulence patterns, out of which 6 patterns (UP3, 4, 5, 6, 11 and 15) were the same as the ones we have observed in our study. Tarchouna et al. (2013) detected 23 various virulence profiles from which 9 profiles (UP1, 4, 7, 8, 9, 10, 12, 14 and 15) existed in this study as well. Among the two studies mentioned and our study, two virulence patterns were exactly similar: UP4 ( *fimH* profile) with 19.53% rate in our study and 23% and 8.88% in the other two studies along with UP15 (no virulence gene) with 3.31% rate in our study and 10% and 8.88% in the two studies mentioned (29, 31).

Recently, the results of several studies in Iran suggest the following antibiotic resistance rates: 69.7%, 78.1%, 54% and 64.7% resistant to trimethoprim-sulfamethoxazole, 43.8%, 31.2% and 43 to norfloxacin, 40.9%, 43.7%, 34% and 61.3% to ciprofloxacin, 53%, 62.5% and 63% to nalidixic acid, 46.2%, 37.5%, 19% and 40% to gentamicin, 25%, 12.5% and 8% to amikacin, 12%, 6.12%, 0 and 0.7% to imipenem (22, 25, 37–39, 41).

Antibiotic resistance to cephalosporins had a wide range of 7.6% to 100%. The highest antibiotic resistance was to cefepime (100%) and the lowest resistance was to cefoxitin (7.6%). Most of the studies reported resistance rates to cephalosporins to be between 50% and 70% (8, 9, 17, 21, 25, 37–39, 42, 43).

Resistance to trimethoprim-sulfamethoxazole (34%) in our study was low, while it holds high rates of 48%, 56.1% and 84% in Kuwait, Mexico and New Delhi, respectively (9, 27, 44).

The highest rate of resistance to fluoroquinolones was 62.3% and the lowest resistance was 11.2%. In the present study, the resistance of *E. coli* isolates to fluoroquinolones (31%) was almost similar to those reported in Iran (34%) and Kuwait (31%) (9, 25, 27, 37, 38, 42).

In all studies, the antibiotic resistance rates to β-lactams and quinolones were > 50%. Only Moue et al. detected a 13.9% resistance to β-lactams in Bangladesh which is similar to our study (13.3%) (8, 37–39, 42–44).

Resistance to aminoglycosides covered a broad spectrum of 2% to 85.24% in all the studies; this resistance was 15.3% in our study which is similar to the findings of Sabir et al. (12.7%) and Pourzare et al. (12.5%) (8, 9, 17, 22, 25, 27, 37–39, 42, 43).

The lowest antibiotic resistance was observed in carbapenems like imipenem and meropenem (4.4%). Several studies reported <% resistance rate for carbapenems, which is similar to the results of our study (23, 27, 38, 39).

Administration of non-standard antibiotics and extensive use of various antibiotics in treating urinary tract infections can lead to different resistances to antibiotics such as increased resistance particularly to cephalosporins in developing countries (38).

If possible, it would be best to collect urine samples from various hospitals in different parts of the city or different cities of the province. Also, investigating a higher number of virulence factor genes is favorable.

## CONCLUSION

Our results indicated that almost half of the isolates carried *fimH* and *aer. fimH* acted as colonization factor and *aer* was important in producing iron for uropathogenic *E. coli*. Understanding the antibiotic resistance in different geographical areas is important; therefore, the most effective antibiotic is chosen to treat urinary tract infections. Meanwhile, it is critical that the antibiotic of choice would not increase the antibiotic resistance. To promote improvement in future studies, it is recommended to collect more *E. coli* isolates from different hospitals and to investigate more virulence factors among these isolates. Furthermore, it is well advised to study the expression of the virulence genes using real time PCR.
